# ULK1 in autophagy: sugar it up

**DOI:** 10.1080/27694127.2022.2139878

**Published:** 2022-10-31

**Authors:** Sheng Yan, Jing Li

**Affiliations:** Beijing Key Laboratory of DNA Damage Response and College of Life Sciences, Capital Normal University, Beijing, China

**Keywords:** autophagy, HNSCC, HPV, O-GlcNAc, ULK1

## Abstract

Autophagy is a highly conserved cellular degradation and recycling process in mammalian cells, which is comprised of three main types: microautophagy, macroautophagy, and chaperone-mediated autophagy (CMA). Autophagy plays a housekeeping role in maintaining cellular homeostasis and therefore can cause many human diseases if it is dysregulated. Our recent paper reported that ULK1 (unc-51 like autophagy activating kinase 1) is glycosylated at S409/S410 and further revealed the biochemical effect in stabilizing this protein.

**Abbreviations**: CMA: chaperone-mediated autophagy; HNSCCs: head and neck squamous cell carcinomas; HPV: human papillomavirus; O-GlcNAc: O-linked β-N-acetylglucosamine; PTM: post-translational modification; ULK1: unc-51 like autophagy activating kinase 1

O-linked β-*N*-acetylglucosamine (O-GlcNAc) modification on serine or threonine residues of proteins, termed O-GlcNAcylation, is an extensive post-translational modification (PTM) that occurs on over 5000 different proteins. Approximately 2-5% of glucose in the human body can be converted to UDP-GlcNAc through the hexosamine biosynthetic pathway, providing the sugar donor for O-GlcNAcylation. There are two enzymes participating in O-GlcNAc modification: OGT (O-linked β-*N*-acetylglucosamine (GlcNAc) transferase), which adds a single UDP-GlcNAc residue to the serine/threonine sites of proteins, and OGA (O-GlcNAcase), which removes UDP-GlcNAc. This process crosstalks with other PTMs and therefore affects a wide range of cellular processes in the human body, such as transcription and translation, cell cycle, nutrient sensing, and stress response. Crucial kinases in autophagy, such as ULK1, have been reported to undergo O-GlcNAcylation and regulate cellular metabolism. O-GlcNAcylation of ULK1 on T635 and T754 promotes the interaction of AMPK with ULK1, thereby upregulating ULK1 activity during autophagy, suggesting that the nutrient-sensing OGT regulates autophagy.

Autophagy also plays a role in viral infection. In the case of human papillomavirus (HPV), two high-risk HPV oncogenes, *E6* and *E7*, bind to *TP53/p53* and *RB1/pRb*, thereby inactivating them and contributing to the development of cervical cancer and head and neck squamous cell carcinomas (HNSCCs). Autophagy helps viral clearance and suppresses the HPV life cycle and tumor development. Meanwhile, when HNSCC cells are infected with HPV, the total protein O-GlcNAcylation levels are significantly increased, indicating that cellular stress response to viral invasion affects protein O-GlcNAcylation modifications.

In our recent study [1], O-GlcNAc levels of ULK1 were found to be elevated in HNSCC UMSCC17B cells in response to HPV infection, suggesting that there could be new O-GlcNAc sites besides T635 and T754. Indeed, subsequent electron transfer dissociation mass spectrometry analysis revealed the new O-GlcNAc site to be S409/S410. We also demonstrate that O-GlcNAcylation at S409/S410 antagonizes phosphorylation at S423, which has been shown previously to subject ULK1 to the CMA-mediated degradation pathway. O-GlcNAcylation hampers the interaction between ULK1 and the CMA chaperone HSPA8/Hsc70 and stabilizes ULK1. Moreover, O-GlcNAcylation promotes ULK1-STX17 interaction and subsequent fusion events between autophagosomes and lysosomes. ULK1 expression is higher in HPV-positive HNSCC as shown by the TCGA database and correlates with the prognosis of HNSCC patients. Taken together, O-GlcNAc glycosylation of ULK1 S409/S410 increases ULK1 stability by inhibiting ULK1 degradation via the CMA pathway, enhances autophagy, and improves the prognosis of HNSCC patients. Our work helps to elucidate a new glycosylation mechanism to regulate ULK1 in the context of HPV infection, thus adjusting the autophagy process to face external and internal conditions.
